# 3,3′-Diindolylmethane Protects against Cardiac Hypertrophy via 5′-Adenosine Monophosphate-Activated Protein Kinase-α2

**DOI:** 10.1371/journal.pone.0053427

**Published:** 2013-01-09

**Authors:** Jing Zong, Wei Deng, Heng Zhou, Zhou-yan Bian, Jia Dai, Yuan Yuan, Jie-yu Zhang, Rui Zhang, Yan Zhang, Qing-qing Wu, Hai-peng Guo, Hong-liang Li, Qi-zhu Tang

**Affiliations:** 1 Department of Cardiology, Renmin Hospital of Wuhan University, Wuhan, China; 2 Cardiovascular Research Institute of Wuhan University, Wuhan, China; 3 The Key Laboratory of Cardiovascular Remodeling and Function Research, Chinese Ministry of Education and Chinese Ministry of Health, Qilu Hospital of Shandong University, Jinan, China; Temple University, United States of America

## Abstract

**Purpose:**

3,3′-Diindolylmethane (DIM) is a natural component of cruciferous plants. It has strong antioxidant and anti-angiogenic effects and promotes the apoptosis of a variety of tumor cells. However, little is known about the critical role of DIM on cardiac hypertrophy. In the present study, we investigated the effects of DIM on cardiac hypertrophy.

**Methods:**

Multiple molecular techniques such as Western blot analysis, real-time PCR to determine RNA expression levels of hypertrophic, fibrotic and oxidative stress markers, and histological analysis including H&E for histopathology, PSR for collagen deposition, WGA for myocyte cross-sectional area, and immunohistochemical staining for protein expression were used.

**Results:**

In pre-treatment and reverse experiments, C57/BL6 mouse chow containing 0.05% DIM (dose 100 mg/kg/d DIM) was administered one week prior to surgery or one week after surgery, respectively, and continued for 8 weeks after surgery. In both experiments, DIM reduced to cardiac hypertrophy and fibrosis induced by aortic banding through the activation of 5′-adenosine monophosphate-activated protein kinase-α2 (AMPKα2) and inhibition of mammalian target of the rapamycin (mTOR) signaling pathway. Furthermore, DIM protected against cardiac oxidative stress by regulating expression of estrogen-related receptor-alpha (ERRα) and NRF2 etc. The cardioprotective effects of DIM were ablated in mice lacking functional AMPKα2.

**Conclusion:**

DIM significantly improves left ventricular function via the activation of AMPKα2 in a murine model of cardiac hypertrophy.

## Introduction

Cardiac hypertrophy is a chronic compensatory condition, in which the heart has suffered from long-term overload. Cardiac hypertrophy can be divided into physiological hypertrophy and pathological hypertrophy [1]. Physiological hypertrophy is a reversible condition that is mainly found in the development of healthy people and pregnant or exercising person. Pathological hypertrophy is mainly characterized by the accumulation of various stimulatory signals (such as heart damage, neurohormonal factors, and aortic stenosis) and is a compensatory response. Initially, in response to a variety of stimuli, myocardial cells increase in size to improve myocardial contractile function and increase myocardial contractility. When the stimulatory factors are sustained, the compensatory mechanism becomes a decompensatory mechanism that eventually leads to heart failure [2], [3]. However, the mechanisms participate in the process of cardiac hypertrophy have not been clearly demonstrated. Up to now, there is no effective method to prevent and treat cardiac hypertrophy. Therapies for cardiac hypertrophy still focus on regulating hemodynamics. Thus, pharmacological interventions targeting the molecular changes involved in cardiac hypertrophy may provide promising approaches for protecting against cardiac hypertrophy and progression to heart failure.

DIM is the major in vivo product derived from the acid-catalyzed condensation of I3C which is a *Brassica* food plant extract material. Studies have found that DIM has a variety of anti-cancer effects, in pancreatic [4], prostate [5] and breast cancer [6]. Moreover, recent studies have shown that DIM has an anti-angiogenic effect. I3C and DIM play anti-angiogenic roles through partly inhibiting of extracellular signal receptor-regulated kinase1/2 (ERK1/2) activity. Compared with I3C, DIM has a stronger role in anti-angiogenesis by inhibiting Akt activity [7]. In addition to participation in the anti-cancer and anti-angiogenic effects, DIM has anti-inflammatory effects. Pervious research has found that in murine macrophages DIM inhibits LPS-induced proinflammatory cytokine release. DIM inhibits the inflammatory response by attenuate the nuclear factor-κB (NF-κB) activity and activator protein 1 (AP-1) signaling pathway [8]. However, the effects of DIM on cardiac hypertrophy and the related signaling mechanisms are not yet clear. Therefore, we aimed to determine whether DIM attenuates cardiac hypertrophy induced by pressure-overload.

In the present study, we show that DIM protects against cardiac hypertrophy by promoting AMPKα phosphorylation. AMPK is a serine/threonine protein kinase that plays an important role in the cardiovascular system [9]. Previous studies have shown that AMPK activation can protect the heart from ischemic injury [10], cell death induced by reactive oxygen species [11] and pressure overload-induced cardiac hypertrophy [12]. In hypertrophic hearts subjected to chronic pressure overload, the activity of both AMPKα1 and AMPKα2 is increased [13]. AMPKα2 was proved to protect against pressure overload-induced ventricular hypertrophy and dysfunction [12]. Increasing number of studies suggest that DIM has various properties, including eliminating free radicals, activating apoptotic signaling pathways, antioxidant and anti-angiogenic effects, and promoting the apoptosis of a variety of tumor cells [4], [7], [14], [15]. DIM can affect mitogen-activated protein kinases (MAPKs), phosphoinositide 3-kinase (PI3K)/Akt and the NF-κB signaling pathway to play anti-cancer, anti-angiogenic and anti-inflammatory roles. The molecular mechanisms of DIM inhibition of the hypertrophic response remain unknown. The purpose of this study were, therefore, to determine whether DIM can attenuate cardiac hypertrophy and fibrosis induced by pressure overload in mice, as well as to identify the molecular mechanisms that may be responsible for its putative effects. In addition, to determine whether the cardioprotective effects of DIM ameliorated in mice lacking functional AMPKα2.

## Materials and Methods

### Materials

Antibodies against total and phosphorylated AMPKα, mTOR, S6, phosphorylated p70 ribosomal protein S6 kinase (p70S6K), phosphorylated translation initiation factor binding protein (4E-BP1) and GAPDH were purchased from Cell Signaling Technology. Antibodies against total p70S6K, total and phosphorylated eukaryotic initiation factor 4E (eIF4E), total translation initiation factor binding protein (4E-BP1) and NRF2 were purchased from Bioworld Technology. Antibodies against Estrogen Related Receptor alpha (ERRα) was purchased from Abcam Inc. The bicinchoninic acid protein assay kit was purchased from Pierce. DIM (>98% purity as determined by HPLC analysis) was purchased from Shanghai Medical Technology Development Co., Ltd. Harmony.

### Animal and Animal Models

All animal procedures were performed in accordance with the Guide for the Care and Use of Laboratory Animals published by the US National Institutes of Health (NIH Publication No. 85-23, revised 1996) and approved by the Animal Care and Use Committee of Renmin Hospital of Wuhan University. Adult male C57/BL6 mice and AMPKα2 knockout mice (C57BL/6 background) (8–10 weeks old) were used in the current study. The diet was based on commonly used diets in rodent intervention studies. Aortic banding (AB) was performed as described previously [16], [17]. Surgery and subsequent analyses were performed in a blinded fashion for all groups. Mice (15–20 per group) received normal feed or feed containing 0.05% DIM (dose: 100 mg/kg/day DIM). After 1 week we subjected the mice to either chronic pressure overload generated by AB or sham surgery as the control group. Mice were randomly assigned into four groups as DIM+sham, DIM+AB, Vehicle+sham, and Vehicle+AB. In a reverse experiment, normal feed containing 0.05% DIM was administered to mice for 7 weeks beginning 1 week after aortic banding surgery to 8 weeks after surgery. Mice were randomly assigned into four groups: DIM(R)+sham, DIM(R)+AB, Vehicle+sham, and Vehicle+AB. AMPKα2 knockout (KO) mice were randomly assigned to four groups: KO+sham, KO+AB, KO+DIM+sham, and KO+DIM+AB. Mouse chow feed containing 0.05% DIM was initiated one week prior to surgery and continued for 4 weeks after surgery. After the mice were killed, the hearts were dissected and weighed to compare heart weight/body weight (HW/BW, mg/g) and heart weight/tibia length (HW/TL, mg/mm) ratios in DIM-treated and vehicle-treated mice.

### Echocardiography and Hemodynamics

Echocardiography was performed by Mylab30CV (ESAOTE S.P.A) with a 10 MHz linear array ultrasound transducer. The LV was assessed in both parasternal long-axis and short-axis views at a frame rate of 50 Hz. End-systole and end-diastole were defined as the phase in which the smallest or largest area of LV was obtained, respectively. Left ventricular end-diastolic diameter (LVEDD) and left ventricular end-systolic diameter (LVESD) were measured from the LV M-mode tracing with a sweep speed of 50 mm/s at the mid-papillary muscle level.

For hemodynamic measurements, mice were anesthetized with 1.5% isoflurane, microtip catheter transducer (SPR-839, Millar Instruments, Houston, TX, USA) was inserted into the right carotid artery and advanced into the left ventricle. The pressure signals and heart rate were recorded continuously with a Millar Pressure-Volume System (MPVS-400, Millar Instruments, Houston, TX, USA), and the data were processed by PVAN data analysis software.

### Histological Analysis

Hearts were excised, washed with saline solution, and placed in 10% formalin. Hearts were cut transversely close to the apex to visualize the left and right ventricles. Several sections of heart (4–5 µm thick) were prepared and stained with H&E for histopathology or PSR for collagen deposition and visualized by light microscopy. For myocyte cross-sectional area, sections were stained for membranes with FITC-conjugated WGA (Invitrogen) and for nuclei with DAPI. A single myocyte was measured with a digital quantitative image digital analysis system (Image Pro-Plus, version 6.0). The outline of 100 myocytes was traced in each group.

### Immunohistochemistry

The procedures were described previously [18]. Heart sections were heated using the pressure cooker method for antigen retrieval. For immunohistochemistry (IHC), the sections were blocked with 3% H_2_O_2_, incubated with anti-NRF2 (Bioworld, rabbit) overnight at 4°C, then incubated with an anti-rabbit EnVisionTM+/HRP reagent, and stained using a DAB detection kit.

### Quantitative Real-time RT-PCR and Western Blotting

Real-time PCR was used to detect those RNA expression levels of hypertrophic and fibrotic markers. Total RNA was extracted from frozen, pulverized mouse cardiac tissue using TRIzol (Roche), and cDNA was synthesized using oligo (dT) primers with the Advantage RT-for-PCR kit (Roche). We performed PCR with LightCycler 480 SYBR Green 1 Master Mix (Roche) and normalized results against glyceraldehyde-3-phosphate dehydrogenase (GAPDH) gene expression. For Western blotting, cardiac tissues were lysed in RIPA lysis buffer. Fifty micrograms of cell lysate was used for SDS/PAGE, and proteins were subsequently transferred to an polyvinylidene difluoride membranes (Millipore). Specific protein expression levels were normalized to the GAPDH protein level for total cell lysate and cytosolic proteins on the same polyvinylidene difluoride membrane. The quantification of Western blots bands was performed with the Odyssey infrared imaging system (Li-Cor Biosciences). The secondary antibodies anti-rabbit IRdye 800 and anti-mouse IRdye 800 (Li-Cor Biosciences) were used at 1∶10000 in Odyssey blocking buffer for 1 hour. The blots were scanned with the infrared Li-Cor scanner, allowing for the simultaneous detection of two targets (antiphospho and anti-total protein) in the same experiment.

### Statistical Analysis

Data are expressed as the means ± SEM. Differences among groups were tested by two-way ANOVA followed by a *post hoc* Tukey test. Comparisons between two groups were performed by unpaired Student’s *t*-test. *P*<0.05 was considered to be significantly different.

## Results

### DIM Attenuated Cardiac Hypertrophy Induced by Pressure Overload in WT Mice

**Figure 1:**
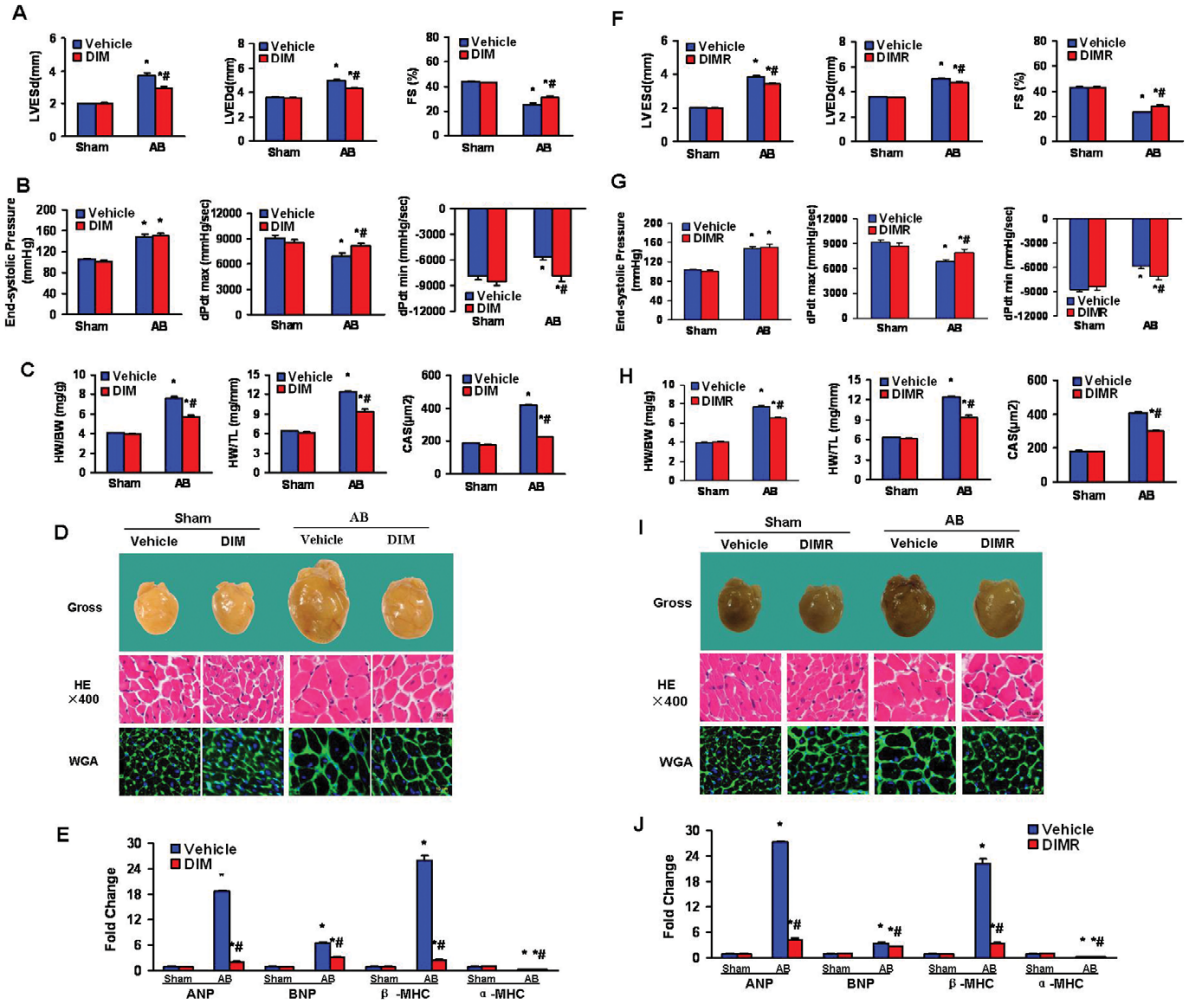
DIM attenuated cardiac hypertrophy induced by pressure overload. (A and B) A, Echocardiography results and B, pressure volume results from four groups of mice at 8 weeks after AB or sham surgery. (C) Statistical results of HW/BW ratio, HW/TL ratio (n = 12) and myocyte cross-sectional areas (n = 100 cells per section) at 8 weeks after AB or sham surgery. (D) Histology: representative gross hearts (top), H&E staining (middle), and WGA–FITC staining (bottom) at 8 weeks after AB or sham surgery. (E) Real-time PCR analysis of hypertrophic markers including ANP, BNP, α-MHC and β-MHC from hearts of mice in the indicated groups (n = 6). (F and G) F, Echocardiography and G, pressure volume results from four groups of mice at 8 weeks after AB or sham surgery in reverse experiments. (H) Statistical results of HW/BW ratio, HW/TL ratio (n = 9) and myocyte cross-sectional areas (n = 100 cells per section) at 8 weeks after AB or sham surgery in reverse experiments. (I) Histology: representative gross hearts (top), H&E staining (middle), and WGA–FITC staining (bottom) at 8 weeks after AB or sham surgery in reverse experiments. (J) Real-time PCR analysis of hypertrophic markers including ANP, BNP, α-MHC and β-MHC, from hearts of mice in the indicated groups in reverse experiment (n = 6). Values are expressed as the mean±SEM. *P<0.05 compared with the corresponding sham group. ^#^ *P<*0.05 *vs* Vehicle+AB group.

Both DIM-treated and vehicle-treated mice were submitted to AB or a sham surgery for 8 weeks to determine whether DIM antagonized and reversed the hypertrophic response to pressure overload. In former and reverse experiments, echocardiographic and pressure-volume (PV) loop analyses of DIM-treated mice showed pressure overload significantly increased LV mass and poor cardiac function in the vehicle-treated group that was limited by DIM replacement. Pressure overload led to increases in left ventricular enddiastole diameter (LVEDD), left ventricular end-systole diameter (LVESD) and decreased fractional shortening (FS) in vehicle-treated mice. LV systolic pressures were similar in the DIM- treated and vehicle-treated mice after 8 weeks of AB, being statistically greater than shams. In the vehicle group, pressure overload significantly decreased maximal LV dP/dt (dp/dt max) and minimum LV dP/dt (dp/dt min, absolute value), both of which were improved by DIM replacement (Figure 1A, B, F, G). DIM replacement normalized these parameters. DIM-treated mice showed attenuated cardiac hypertrophy after 8 weeks of AB with improvements in heart weight/body weight, heartweight/tibia length, and cardiomyocyte cross sectional area (Figure 1C, H). The inhibitory effect of DIM on cardiac hypertrophy was confirmed by the morphology of the gross hearts, hematoxylin-eosin (H&E) staining, and wheat germ agglutinin (WGA) staining (Figure 1D, I).

Quantitative increases in cardiac mass after pressure overload are always accompanied by the re-expression of a fetal gene program. Under baseline conditions, atrial natriuretic peptide (ANP), B-type natriuretic peptide (BNP), and β-myosin heavy chain (β-MHC) was strikingly suppressed in DIM-treated after pressure overload, accompanied by the upregulation of α-myosin heavy chain (α-MHC). (Figure 1E, J). Collectively, these data suggested DIM that impaired cardiac hypertrophy and protected cardiac function after pressure overload.

### DIM Promoted the Phosphorylation of AMPKα and Attenuated mTOR Signaling Activation Induced by Pressure Overload

**Figure 2:**
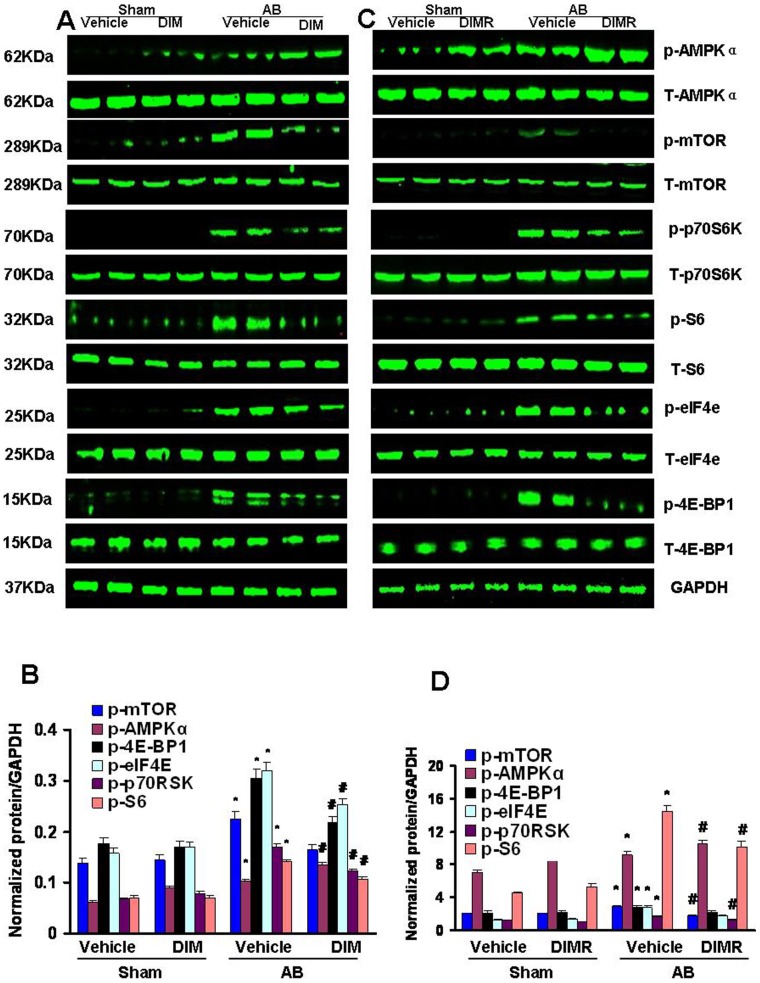
The effect of DIM on AMPKα and mTOR/p-70S6K signaling. (A and B) The protein levels of phosphorylated and total AMPKα, mTOR, p70S6K, S6, eIF4e and 4E-BP1 in mice from indicated groups. A, Representative Western blots. B, Quantitative results (n = 6). (C and D) The protein levels of phosphorylated and total AMPKα, mTOR, p70S6K, S6, eIF4e and 4E-BP1 in mice from indicated groups in reverse experiments (n = 6). C, Representative Western blots. D, Quantitative results. Values are expressed as the mean±SEM. *P<0.05 compared with the corresponding sham group. ^#^ *P<*0.05 *vs* Vehicle+AB group.

To explore the molecular mechanisms of the antihypertrophic actions of DIM, we analyzed protein extracts by Western blotting. In vehicle-treated mice, pressure overload significantly increased the cardiac amounts of p-AMPKα, increases that were promoted after DIM-treated. Phosphorylation of mTOR, p70S6K, S6, eIF4e and 4E-BP1, the known downstream targets of AMPKα, were also increased after 8 weeks of AB. However, they were blocked in DIM-treated hearts (Figure 2A, B, C, D). Collectively, these data suggest that DIM significantly inhibits cardiac hypertrophy through directly stimulating p-AMPKα activity and inhibiting the mTOR/p70S6K signaling pathway.

### DIM Inhibited the Fibrosis Induced by Pressure Overload in WT Mice

**Figure 3:**
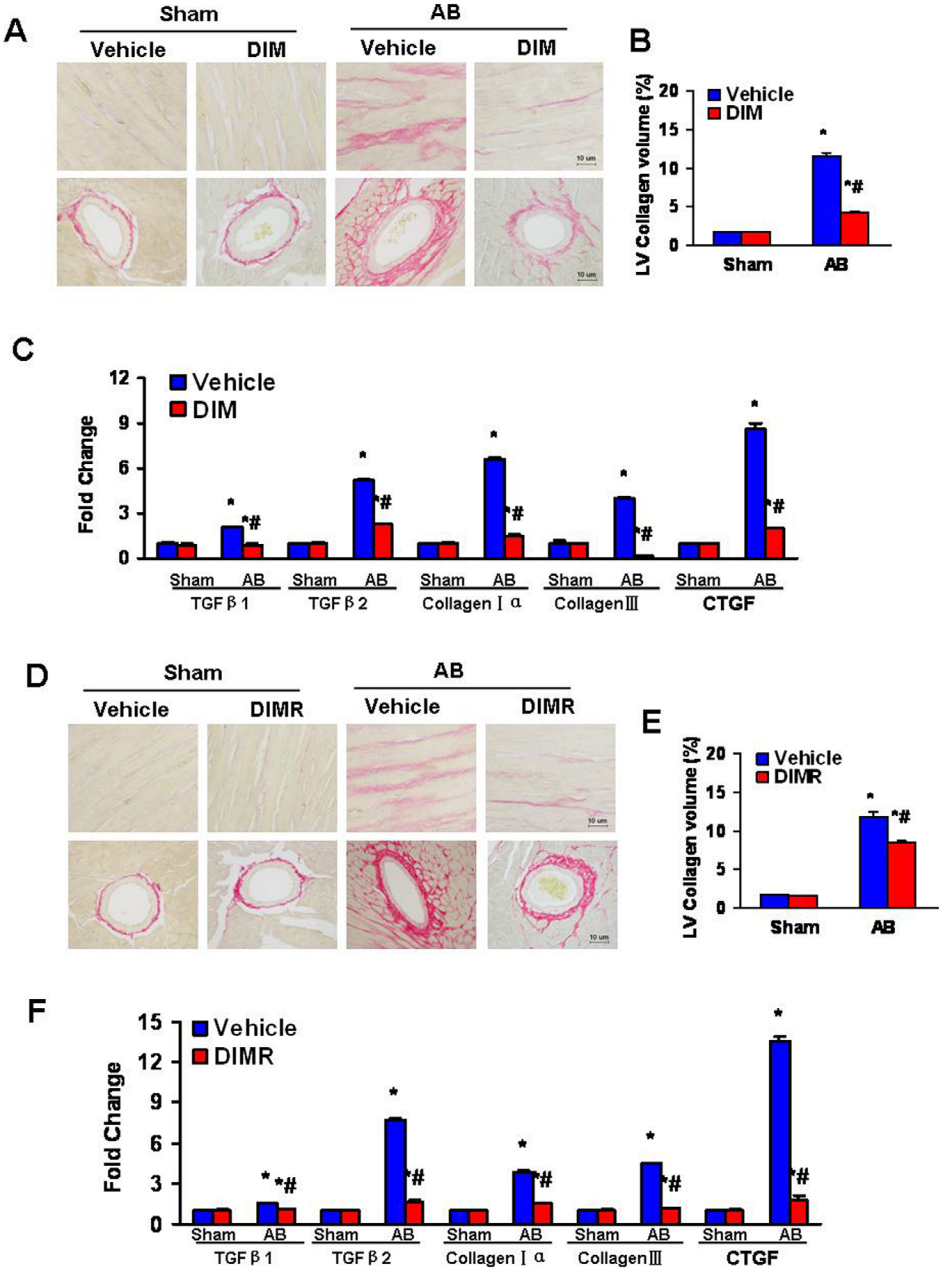
DIM blocked the fibrosis induced by pressure overload. (A) Representative images of PSR staining form indicated groups. (B) Quantitative analysis of left ventricle interstitial collagen volume fraction in indicated groups (n = 6). (C) Real-time PCR analysis of the mRNA expression of TGF-β1, TGF-β2, collagen Iα, col1agen β and CTGF in the myocardium obtained from indicated groups (n = 6). (D) Representative images of PSR staining form indicated groups. (E) Quantitative analysis of left ventricle interstitial collagen volume fraction in indicated groups in reverse experiments (n = 6). (F) Real-time PCR analysis of the mRNA expression of TGF-β1, TGF-β2, collagen Iα, col1agen β and CTGF in the myocardium obtained from indicated groups in reverse experiments (n = 6). The results were reproducible in three separate experiments. Values are expressed as mean±SEM. *P<0.05 compared with the corresponding sham group. ^#^ *P<*0.05 *vs* Vehicle+AB group.

To determine the extent of left ventricular interstitial fibrosis in the heart, in both former and reverse experiments, paraffin-embedded slides were stained with picrosirius red (PSR). Perivascular and interstitial fibrosis was detected in both vehicle-treated and DIM-treated mice, but the extent of cardiac fibrosis was markedly reduced in DIM treated mice (Figure 3A, B, D, E). Subsequent analysis of mRNA expression levels of known mediators of fibrosis, including transforming growth factor β1 (TGF-β1), transforming growth factor β2 (TGF-β2), procollagen type Iα (col1agen Iα), procollagen type β (col1agen β) and connective tissue growth factor (CTGF), demonstrated a blunted response in DIM-treated mice after 8 weeks of AB (Figure 3C, F).

### DIM had no Protective Effect on Cardiac Hypertrophy Induced by Pressure Overload in AMPKα2 Knockout Mice

**Figure 4:**
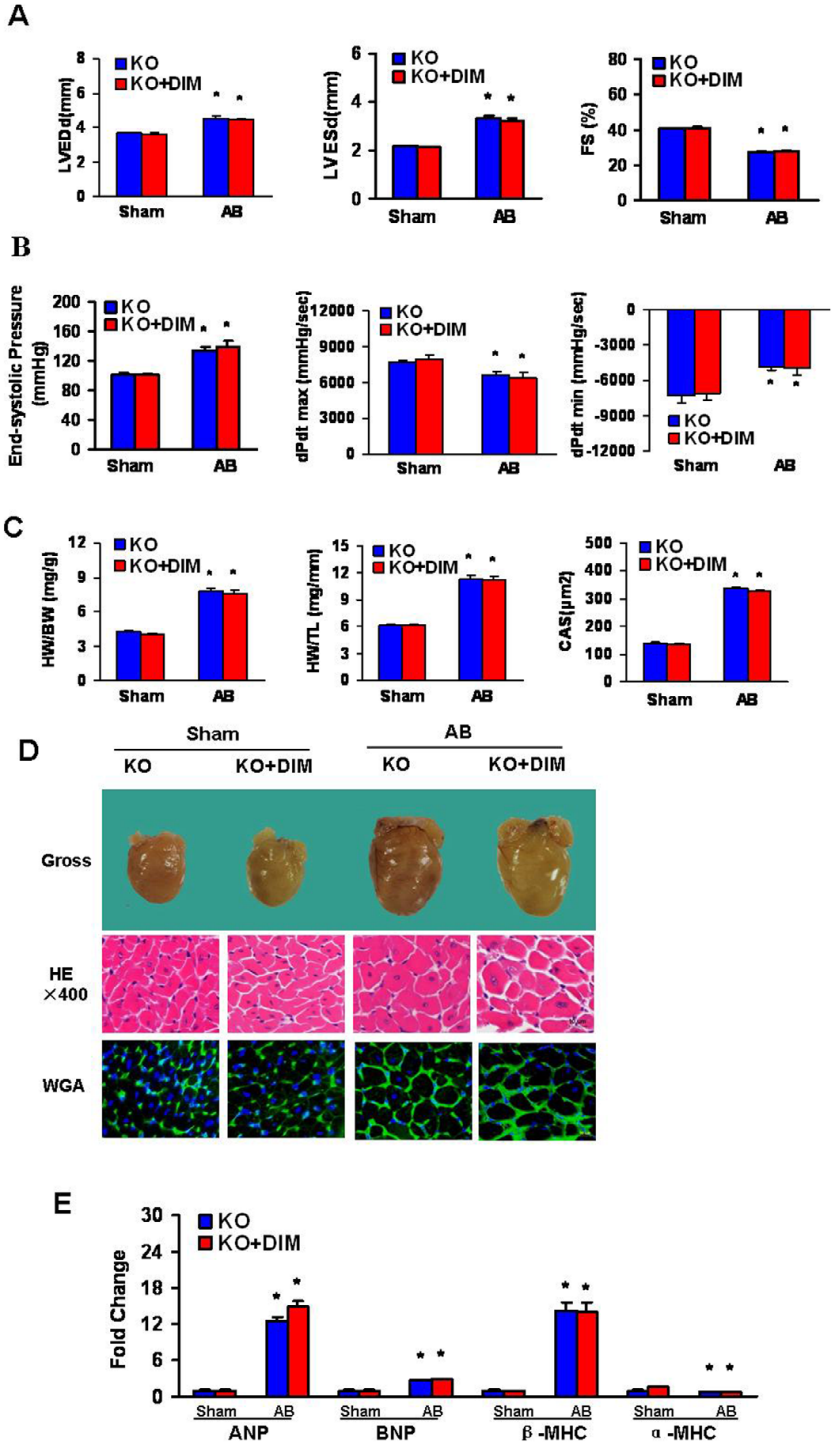
DIM inhibited cardiac hypertrophy by targeting AMPKα2. (A and B) Echocardiography and pressure volume results from four groups of mice at 4 weeks after AB or sham surgery. (C) Statistical results of the HW/BW ratio, HW/TL ratio (n = 12) and myocyte cross-sectional areas (n = 100 cells per section) at 4 weeks after AB or sham surgery. (D) Histology: representative gross hearts (top), H&E staining (middle) and WGA–FITC staining (bottom). (E) Real-time PCR analysis of hypertrophic markers including ANP, BNP, α-MHC and β-MHC, from hearts of mice in the indicated groups (n = 6). Values are expressed as mean±SEM. *P<0.05 compared with the corresponding sham group.^ #^ *P<*0.05 *vs* KO+AB group.

The above data, which shows the ability of DIM to inhibit cardiac hypertrophy, suggest that synaptic activity may be modulated by AMPKα. To examine whether AMPK was critical for the effect of DIM in the hypertrophic response to pressure overload, AMPKα2 knockout (KO) mice were subjected to AB surgery or sham surgery. After 4 weeks of AB, AMPKα2 deficiency significantly more impairment of cardiac function, as demonstrated by a significant increase in LVEDD and LVESD, and a greater reduction of systolic fractional shortening as compared with Sham mice. Pressure-volume loop analysis further revealed that AMPKα2 deficiency worsened hemodynamic dysfunction in response to AB. Moreover, DIM treatment did not prevent the development of adverse cardiac remodeling or ventricular dysfunction in AMPKα2 KO mice (Figure 4A, B). Pressure overload-induced increased HW/BW, HW/TL and myocyte cross-sectional (Figure 4C), according with the morphology of the gross hearts, hematoxylin-eosin staining, and wheat germ agglutinin staining (Figure 4D) However, these measures were not improved after DIM-treated. In addition, the mRNA levels of ANP, BNP and β-MHC, which were the induction of hypertrophic markers, were greatly escalated and α-MHC was decreased in both DIM-treated and vehicle-treated KO mice in response to AB (Figure 4E). These results suggest that DIM could negatively regulate the extent of cardiac hypertrophy through AMPKα2 in response to pressure overload.

### DIM Lost Protective Property against the Fibrosis Induced by Pressure Overload in AMPKα2 Knockout Mice

**Figure 5:**
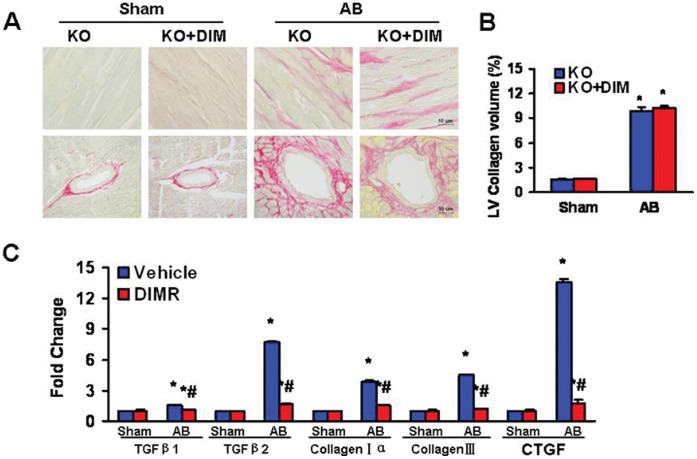
AMPKα2 is necessary in the anti-fibrotic effect of DIM. (A) Representative images of PSR staining form indicated groups. (B) Quantitative analysis of left ventricle interstitial collagen volume fraction in indicated groups (n = 6). (C) Real-time PCR analysis of the mRNA expression of TGF-β1, TGF-β2, collagen Iα, col1agen β and CTGF in the myocardium obtained from indicated groups (n = 6). The results were reproducible in three separated experiments. Data are expressed as means±SEM. *P<0.05 compared with the corresponding sham group.^ #^ *P<*0.05 *vs* KO+AB group.

Paraffin-embedded slides were stained with picrosirius red (PSR) to explore whether AMPK was also critical for the effect of DIM on fibrosis in the heart. Perivascular and interstitial fibrosis was detected in both DIM-treated and vehicle-treated KO mice subjected to AB or sham surgery, but the extent of cardiac fibrosis was markedly enhanced in both AB group and no difference between them (Figure 5A, B). Subsequent analysis of mRNA expression levels of TGF-β1, TGF-β2, col1agen Iα, col1agen β and CTGF, which are responsible for cardiac fibrosis, showed the similar results (Figure 5C).

### Role of DIM in the Regulation of Cardiac Oxidative Stress after Pathological Pressure Overload

**Figure 6:**
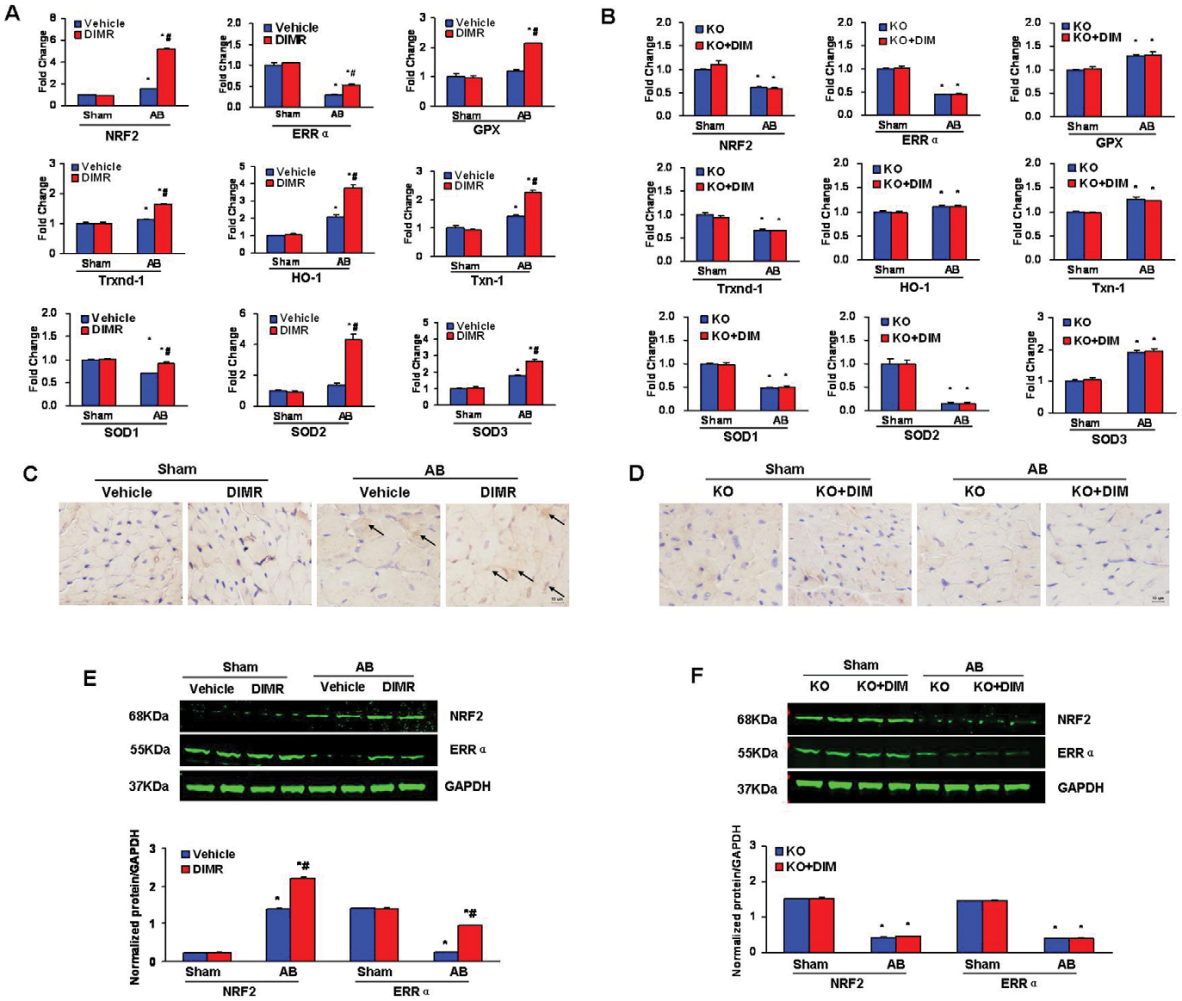
DIM inhibited Cardiac Oxidative Stress through AMPKα2. (A and B) Real-time PCR analysis of the mRNA expression of ERRα, Nrf2 and its downstream genes including GPx, HO-1, Txn-1, Txnrd-1, SOD1, SOD-2 and SOD-3 in the myocardium obtained from indicated groups (n = 6). A, in WT mice and B, in AMPKα2 KO mice. (C and D) Representative immunohistochemial staining of Nrf2 protein expression in WT mice and AMPKα2 KO mice after AB. (E and F) The protein levels of the cardiac expression of Nrf2 and ERRα in the myocardium obtained from indicated groups (n = 6). E, in WT mice and F, in AMPKα2 KO mice. Top, Representative Western blots; bottom, Quantitative results. *P<0.05 compared with the corresponding sham group. ^#^ *P<*0.05 *vs* Vehicle+AB/KO+AB group.

Resent studies shown that DIM induces the transactivation of Nrf2 in cultured murine fibroblasts [19], while AMPKα2 regulates expression of estrogen-related receptor-alpha (ERRα, a transcriptional factor often affects mitochondrial antioxidants and energy metabolism) [20]. Thus, we detect the myocardial oxidative stress markers and expression of NRF2 and ERRα etc after DIM-treated in both wild type and AMPKα2 KO mice. Pressure overload increased myocardial expression of ERRα, Nrf2 and its downstream genes, including glutathione peroxidase (GPx), heme oxygenase 1 (HO-1), thioredoxin-1 (Txn-1), thioredoxin reductase (Txnrd-1), superoxide dismutase (SOD)-1, SOD-2, and SOD-3 mRNAs in DIM-treated WT mice (Figure 6A) but not in AMPKα2 KO mice (Figure 6B). Nuclear translocation of Nrf2 proteins was dramatically enhanced in the cardiomyocytes of DIM-treated WT hypertrophied hearts (Figure 6C) but not in AMPKα2 KO hearts after AB (Figure 6D). The protein expression of Nrf2 and ERRα were consistent with the mRNAs expreesion (Figure 6E, F). These results indicate that Nrf2 and ERRα expression and activity are enhanced by DIM partly through AMPKα2 in the process of maladaptive responses to the sustained hemodynamic stress.

## Discussion

The results from our study demonstrate that DIM protects against cardiac hypertrophy both in former and reverse experiments. The protective role of DIM in cardiac hypertrophy is mediated by direct interruption of AMPK-dependent mTOR signaling. The biological effects of DIM are also robust enough to reverse established cardiac hypertrophy induced by chronic pressure overload. More importantly, the cardioprotective effects of DIM ameliorated in mice lacking functional AMPKα2.

As a traditional Chinese medicine, DIM is widely used in combating against various diseases, such as anti-cancer, anti-angiogenic and anti-inflammatory effects, involved in affecting MAPKs, PI3K/Akt and the NF-κB signaling pathway [7], [8]. In the present study, we have demonstrated that DIM not only attenuated cardiac hypertrophy in response to hypertrophic stimuli but also improved cardiac performance and reduced chamber dimensions. Another main finding of this study is that DIM could reverse pre-established cardiac hypertrophy and dysfunction induced by chronic pressure overload, which is of markedly clinical relevance.

The biochemical mechanism by which DIM mediates its antihypertrophic effects remains elusive. AMPK is an important regulator of cardiac metabolism [21]. AMPK signaling appears to have comprehensive effects in cardiovascular health and disease [22]. Previous study has demonstrated that *in vitro* AMPK activation is a key mediator of the changes in substrate utilization during cardiac ischemia and functions to maintain energy homeostasis, cardiac function and myocardial viability [10]. *In vivo* studies have shown that AMPKα2 plays a significant role in regulating pressure-overload induced LV remodeling [12].Our data demonstrate that DIM increases AMPKα phosphorylation. However, the cardioprotective actions of DIM ameliorated in AMPKα2 deficiency mice. AMPKα2 disruption aggravated cardiac hypertrophy, fibrosis, and dysfunction after AB in both vehicle-treated and DIM-treated groups. These results suggest that the chronic activation of AMPKα2 during the development of cardiac hypertrophy is a primary mechanism mediating the beneficial actions of DIM.

We first found DIM interferes with the activation of AMPKα phosphorylation. Although AMPK activation is generally linked to degrading processes, this action seems to be largely indirect via mammalian target of rapamycin (mTOR) inhibition [23]. mTOR plays a significant role in the development of cardiovascular disease [24]. It is well known that the main downstream targets of mTOR are the S6 kinases (p70/85 and p54/56), the translation initiation factor binding protein (4E-BP1) [25], [26], which is a repressor of eukaryotic translation initiation factor 4E (eIF4E) [27], [28]. Importantly, previous studies have shown mTOR or its target(s) plays an important role in cardiac hypertrophy [29] and mTOR is required for the development of cardiac hypertrophy induced by rising blood pressure in spontaneously hypertensive rats [30]. In line with results from previous studies, we observed a significant increase in the phosphorylated levels of mTOR, p70S6K, S6, 4E-BP1 and eIF4E in the hypertrophic hearts of AB mice. When DIM was administered, the phosphorylation of them were markedly blocked. These findings indicate that the inhibitory effects of DIM on cardiac hypertrophy are mediated through mTOR signaling.

Fibrosis is another classical feature of pathological hypertrophy, which is characterized by the expansion of the extracellular matrix due to the accumulation of collagen [31]. Cardiac fibrosis plays a major role in the development of abnormal myocardial stiffness and ventricular dysfunction in response to pathological stimuli [32]. Thus, it is important to explore the mechanisms that stimulate collagen deposition in the heart and define approaches to limit these processes. As shown here, DIM-treated significantly attenuated cardiac fibrosis after pressure overload in WT mice and AMPKα2 deficiency mice. In current study, we found that DIM could downregulate mRNA expression levels of known mediators of fibrosis including TGF-β1, TGF-β2, collagen Iα, col1agen β and CTGF were blunted in WT mice after AB. However, the mRNA expression levels of fibrosis mediators were markedly enhanced in both AMPKα2 KO AB groups and no difference was found between them. These results show that DIM could inhibit the fibrosis induced by pressure overload, primarily through AMPKα2.

Oxidative stress has been identified as one of the key contributing factors in the development of cardiac hypertrophy [33]. Recent research has suggested DIM induced Nrf2 transactivation [19], which is a protective regulator that prevents the maladaptive cardiac remodeling and heart failure associated with a sustained pathological hemodynamic stress via at least partly the suppressing of oxidative stress [32]. Loss of Nrf2 sensitizes cardiomyocytes and cardiac fibroblasts to oxidative stress-mediated cell death [34]. In present study, we found DIM increased myocardial expression of Nrf2 and its downstream genes in WT mice but not in AMPKα2 KO mice after AB. In addition, we detected DIM-treated resulted in a significant increase of myocardial ERRα at both mRNA and protein levels in WT mice but not in AMPKα2 KO mice after AB. Similar to our observations in AMPKα2 KO mice, previous study found that the reduced ERRα expression in the AMPKα2 KO mice contributed to the dysregulation of only some of the energy metabolism related genes [20].

In conclusion, our present study showed for the first time that DIM can not only prevent the development of cardiac hypertrophy but also reverse established cardiac hypertrophy by regulating the AMPKα and mTOR. The cardioprotective effects of DIM were ameliorated in mice lacking functional AMPKα2. This study is highly relevant to the understanding of the inhibitory effect of DIM on cardiac hypertrophy and related molecular mechanisms. Our observations revealed new insight into the pathogenesis of cardiac remodeling and may have considerable implications for the development of strategies for the treatment of cardiac hypertrophy and heart failure through the application of DIM. Additional studies are necessary to elucidate the potential clinical use of DIM.
